# Integrated Lipidomics and Metabolomics Analysis of Tears in Multiple Sclerosis: An Insight into Diagnostic Potential of Lacrimal Fluid

**DOI:** 10.3390/ijms20061265

**Published:** 2019-03-13

**Authors:** Ilaria Cicalini, Claudia Rossi, Damiana Pieragostino, Luca Agnifili, Leonardo Mastropasqua, Maria di Ioia, Giovanna De Luca, Marco Onofrj, Luca Federici, Piero Del Boccio

**Affiliations:** 1 Department of Pharmacy, University ‘‘G. d’Annunzio’’ of Chieti-Pescara, 66100 Chieti, Italy; ilaria.cicalini@unich.it (I.C.); piero.delboccio@unich.it (P.D.B.); 2 Analytical Biochemistry and Proteomics Laboratory, Centre on Aging Sciences and Translational Medicine (Ce.S.I-MeT), University ‘‘G. d’Annunzio’’ of Chieti-Pescara, 66100 Chieti, Italy; dpieragostino@unich.it (D.P.); lfederici@unich.it (L.F.); 3 Department of Medical, Oral and Biotechnological Sciences, University ‘‘G. d’Annunzio’’ of Chieti-Pescara, 66100 Chieti, Italy; 4 Opthalmic Clinic, Department of Medicine and Aging Science, “G. d’Annunzio” University of Chieti-Pescara, 66100 Chieti, Italy; l.agnifili@unich.it (L.A.); leonardo.mastropasqua@unich.it (L.M.); 5 Unit of Neurology Ss Annunziata Hospital, 66100 Chieti, Italy; maria.diioia@unich.it (M.d.I.); gio.deluca05@yahoo.com (G.D.L.); marco.onofrj@unich.it (M.O.); 6 Department of Neuroscience, Imaging and Clinical Sciences, “G. d’Annunzio” University of Chieti-Pescara, 66100 Chieti, Italy

**Keywords:** metabolomics, lipidomics, tears, biomarkers, amino acids, acylcarnitines, multiple sclerosis

## Abstract

Metabolomics based on mass spectrometry represents an innovative approach to characterize multifactorial diseases, such as multiple sclerosis (MuS). To date, the most important biomarker source for MuS diagnosis is the cerebrospinal fluid. However, an important goal for research is to identify new molecules in more easily accessible biological fluids. A very interesting biofluid in MuS is represented by tears, considered as an intermediate fluid between the cerebrospinal fluid and serum. In this work, we developed a merged strategy for the analysis of lipids containing choline by Liquid Chromatography coupled to Tandem Mass Spectrometry (LC-MS/MS), as well as for the targeted analysis of free carnitine, acylcarnitines and aminoacids by direct infusion mass spectrometry. Samples for both metabolomics and lipidomics approaches were obtained in a single extraction procedure from tears of patients affected by MuS and healthy controls. Tear lipidomics showed 30 phospholipids significantly modulated and, notably, many sphingomyelins resulted lower in MuS. Moreover, the metabolomics approach carried out both on tears and serum highlighted the diagnostic potential of specific aminoacids and acylcarnitines. In conclusion, the metabolic profiling of tears appears to reflect the pathological conditions of the central nervous system, suggesting that the molecular repository of tears can be considered as a source of potential biomarkers for MuS.

## 1. Introduction

Multiple sclerosis (MuS) is a demyelinating disease with an autoimmune pathogenesis. The exogenous, environmental, and genetic factors are considered the main agents responsible for MuS development. Despite efforts, it is still difficult to achieve an early and definite diagnosis that allows the establishment of an early treatment. Thus, the discovery of new biomarkers is necessary [1]. It has been reported that lipids play a role in the autoimmune process, and new evidence shows lipid metabolism alterations in the central nervous system (CNS) of MuS subjects [2,3]. Indeed, our previous works demonstrated that metabolomics and lipidomics approaches may be useful in identifying metabolic alterations in sera and in cerebrospinal fluid (CSF) from MuS patients [4,5]. In a previous work by Gonzalo et al. [6], several discriminant metabolites were identified between MuS and non-MuS patients through untargeted metabolomics and lipidomics approaches. It was also demonstrated that fatty acid metabolism may be involved in the immunological response and inflammation through free carnitine (C0) and acyl-carnitines (ACs), which regulate the energy production in immune cells [7]. Acetyl-carnitine has also been proposed as a therapeutic agent in several neurodegenerative diseases. The therapeutic rationale in MuS derives from the demonstration of a reduction of nitroxidative stress in the CSF of active MuS patients treated with acetyl-carnitine [8]. Our recent work reported data about metabolic regulation in MuS both in CSF [5] and serum [9], showing many potential biomarkers for MuS. Nevertheless, to date, the most important biomarkers for MuS diagnosis remain the oligoclonal bands (OCBs) in CSF and link index [10,11] associated to magnetic resonance imaging (MRI). CSF is certainly the most informative body fluid for the study of the pathological processes occurring in the CNS, although it is obtained through an invasive procedure. Therefore, one main goal of biomarker research in MuS would be the identification of novel distinctive molecules in more easily accessible biological fluids. In this light, it is already known that tears may represent a precious resource for studying MuS [12,13], since the eye can be considered an extension of the CNS, hence reflects its physiopathological condition [14].

Tear film represents an elective structure for the investigation of ocular and non-ocular disorders since it is easily accessible [15,16,17,18]. Recently, “omics” technologies have been used to identify molecular alterations in tears [19,20] associated to different disorders, not only eye-related. Tear lipidomics approaches were used to characterize dry eye syndrome [21] and Meibomian gland dysfunction [22]. In particular, L-carnitine and short chain esters, as well as steroids levels [16], have been already found altered in tears from dry eye patients [23]. Tears have also been studied in MuS: different authors [24,25] found OCBs in tears of patients with MuS and in radiologically isolated syndrome (RIS) patients. Notably, CSF sensitivity is very similar (55%) to tears sensitivity (50%). More recently, Lebrun et al. have confirmed this evidence, suggesting that the detection of tear OCB may replace the CSF one in patients with RIS, especially in consideration of the follow-up needs [26]. The main limitation of this biofluid is represented by the low sample amount, which often restricts the number of screenable molecules. In this work we developed a new LC-MS/MS strategy for the analysis of polar lipids, free carnitine (C0), acylcarnitines (ACs) and amino acids (AAs) in a single extraction procedure from tears, allowing the analysis of different classes of biomolecules from the same sample. The developed method was used to analyse tears samples from a pilot cohort of patients affected by MuS and healthy controls (HCs), demonstrating the potential of tears as a source of biomarkers for complex diseases, and opening the opportunity for large population screening.

## 2. Results

### 2.1. Tears Metabolites Extraction from Schirmer’s Strip

**Figure 1:**
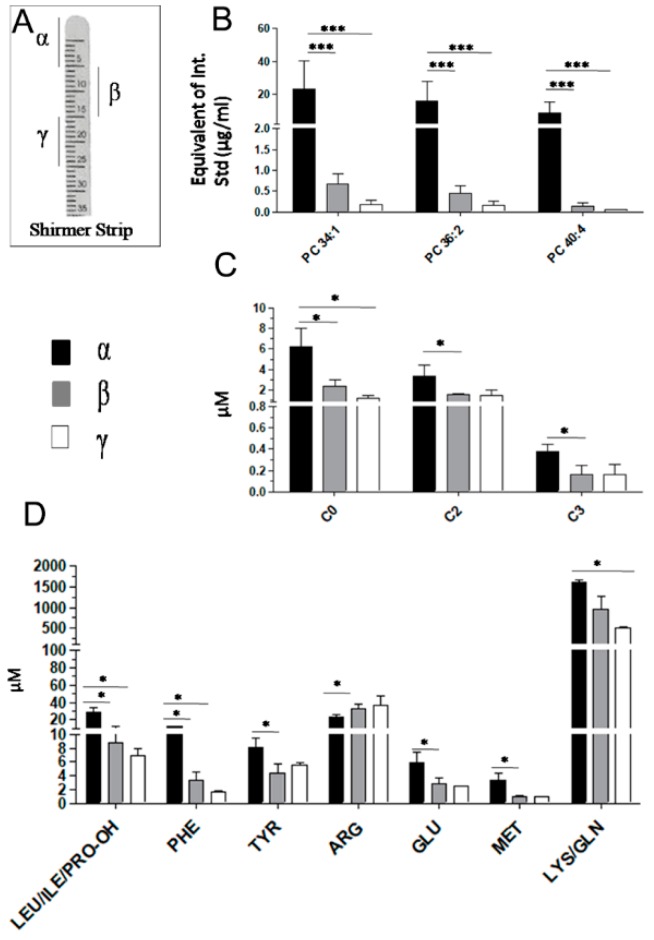
Study of metabolites distribution in paper strip. (**A**) Figure shows the amount of metabolites measured in three sections of 10 mm called α, β and γ to determine whether the metabolites are evenly distributed along the strip. (**B**) Histograms show the amount of PC 34:1, PC 36:2 and PC 40:4 expressed as equivalent of Internal Standard (µg/mL) along the paper strip. (**C**,**D**) Panels show the concentration of free carnitine (C0), acyl carnitines (C2, C3) and amminoacids (Leu/Ile/Pro, Phe, Tyr, Glu, Met, Lys/Gln/Arg) in the three sections of the Shirmer’s paper strip. * means *p* < 0.05, ** means *p* < 0.01 and *** means *p* < 0.001 at the Student’s *t*-test.

Following the procedure described in the methods section, we collected three tears samples from an HC over three different days. The Schirmer’s paper strips were cut into three sections of 10 mm called α, β and γ (Figure 1 Panel A) to determine whether the metabolites were evenly distributed along the strip. Results are reported in Figure 1 Panels B-D. In particular, we selected phosphatidylcholines PCs (PC 34:1, PC 36:2 and PC 40:4) in Panel B, C0, ACs (C2, C3) in Panel C and AAs (Leu/Ile/Pro, Phe, Tyr, Glu, Met, Lys/Gln) in Panel D for the results to be retained in the first section (α) of the paper strip. Only Arg shows an opposite trend (Figure 1 Panel D). These data demonstrate the need to use the whole Schirmer’s strip to carry out a homogeneous extraction procedure for each tear sample.

**Figure 2:**
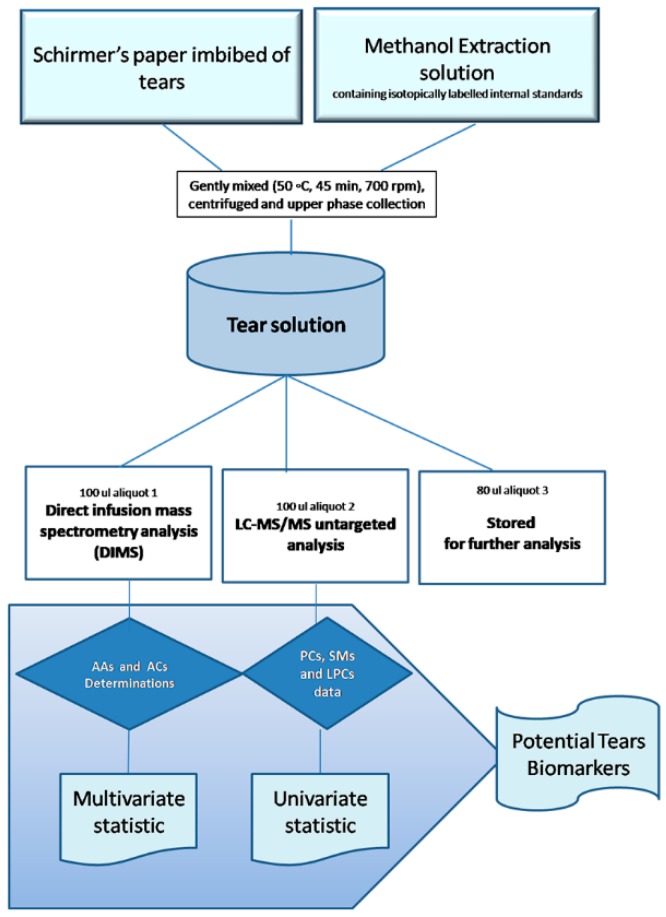
Workflow of tear lipidomics and metabolomics strategy. The scheme summarizes the extraction procedure phases for both lipidomics LC-MS/MS analysis and for targeted metabolomics DIMS analysis.

Subsequently, we quantified the forty-two metabolites in thirty-three Schirmer’s strips imbibed with different millimetres of tears (from 1 mm to 35 mm). Linear regressions between the amount of each metabolite and the millimetres of tears obtained in 5 min of collection (95% confidence interval) show (Table 1) no correlation between the mm of imbibed strip and the concentration of analytes (not significant R^2^). Based on this evidence, we optimized a single extraction procedure of the entire Schirmer’s strip (35 mm) for metabolomic and lipidomic analysis, independently of the imbibed mm of the strip, as reported in the workflow (Figure 2).

### 2.2. Tears Lipidomics by LC-ESI-MS/MS

**Figure 3:**
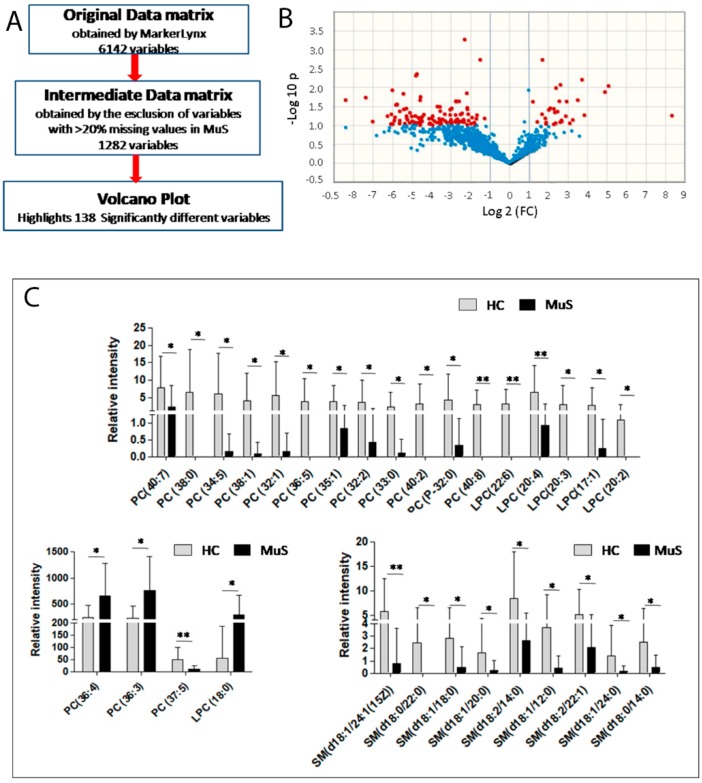
Lipidomic profiling in tears and sera from MuS patients. (**A**) Workflow description for tears lipidomics data processing. (**B**) Figure shows the Volcano Plot: a combination of fold change (FC) and *t*-tests. The x-axis is log2 FC and the Y-axis is log10 (*p*-value) obtained by non-parametric test. The red dots are significant with the -log10 (*p*-value) > 1. The red dots on the right represent the lipids with higher levels in MuS patients, while the dots on the left are the lipids with lower levels in MuS tears in respect to HCs. (**C**) In the histograms the relative abundance of the tear identified significantly different phospholipids (divided into 15 PCs, 6 LPCs and 11 SMs) is reported. In the grey bars the mean and SD of control group are reported, while in black bars the mean and SD of the MuS patients are reported. * means *p* < 0.05 and ** means *p* < 0.01 at the Student’s *t*-test or Mann Whitney U-test.

In a previous paper, we have already discussed the goodness of this approach in studying phospholipids in serum samples of MuS patients, even in pregnancy [4]. Here, phospholipids from 12 MuS patients and 21 HCs were analyzed from tears, focusing on the acquisition of PCs and a sphingomyelins (SMs) profile. Data revealed 6142 variables related to PCs and SMs molecular species. Lipidomics data were processed following the procedure schematized in Figure 3 Panel A. In particular, the dataset was reduced, taking into account only lipid signals present in at least 20% of the analyzed group in order to eliminate most of the useless variables. After the pre-processing, the number of variables dramatically decreased to 1282. These phospholipid signals were then subjected to non-parametric univariate statistical analysis with the aim to highlight specific alterations of lipid patterns in MuS. Figure 3 Panel B shows the volcano plot, reporting the main discriminant PCs and SMs between MuS and HC tear samples. The Mann Whitney U-test highlights 138 significantly different signals between the two groups. The differential signals were identified using Lipidmaps and Human Metabolome Databases (HMDB), obtaining 32 phospholipids (divided into 15 PCs, 6 lysoPCs LPCs and 11 SMs). The histograms in Figure 3 Panel C show the relative intensity of the PCs, LPCs and SMs species regulated in MuS. Interestingly, most of the differential lipids found in tears result lower in MuS, according to our previously published lipidomics data in CSF [27].

### 2.3. Targeted Metabolomics by Direct Infusion Mass Spectrometry (DIMS) Analysis

#### 2.3.1. Tears DIMS Analysis

The targeted metabolomics approach using DIMS was employed for the analysis of tear samples collected on Schirmer paper strips. The raw data matrix, obtained by NeoLynx software (Waters, Milford, MA, USA), was used to perform a multivariate statistical analysis, in order to highlight a specific metabolic pattern able to distinguish MuS tears from HC ones.

**Figure 4:**
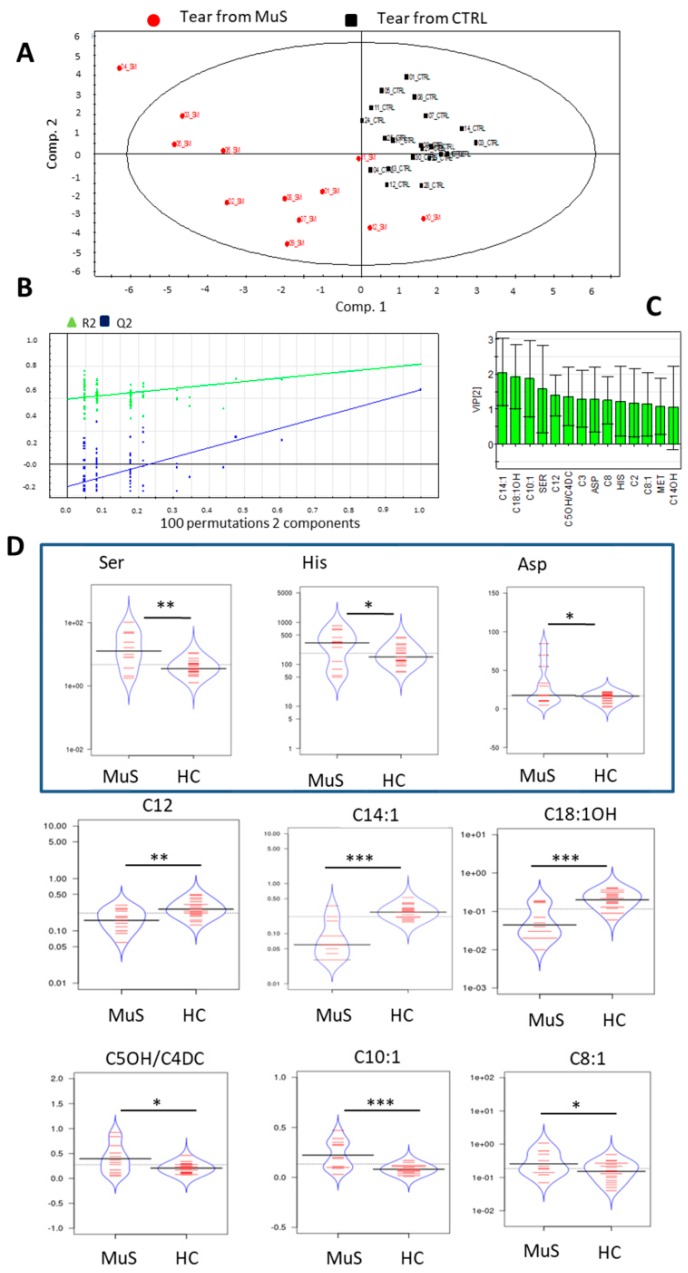
Tears metabolomics investigation by PLS-DA (**A**) Panel shows the Scores scatter plot calculated on two components using 43 tear metabolite concentrations. The PLS-DA) are used to classify tears from MuS patients (symbolized as red dots) from HC (indicated as black squares) obtained by R2Y = 0.77 and Q2(cum) = 0.586. (**B**) Panel shows the validate model plots for tears PLS-. The plot shows on the vertical axis the R2 and Q2 for the original model (far to the right) and of the Y-permutated models further to the left. (**C**) Panel shows the VIP plots related to the PLS-DA of tear metabolomics investigation. (**D**) We report the distribution of the significantly different AAs and ACs in the two analyzed groups, visualized as the bean plots. In these plots the polygon shape (in blue line) represents the density trace computed using a log-transformation of each variable, and next to that, a scatter plot shows all individual measurements (red line). * means *p* < 0.05, ** means *p* < 0.01 and *** means *p* < 0.001 at the Student’s *t*-test.

The mean abundance for tear AAs and ACs, as determined by DIMS analysis, was reported in Table S1. The above mentioned metabolites (Table S1) were used to build the scores scatter plot calculated on two components, as shown in Figure 4 Panel A. In particular, 17 AAs and 23 ACs were used to re-classify MuS patients from HCs through Partial Least Square Discriminant Analysis (PLS-DA). The multivariate analysis shows an unambiguous separation between the two clinical groups (MuS patients are symbolized by red dots, while HCs are indicated by black squares), thus obtaining a PLS-DA model with R2Y = 0.77 and Q2(cum) = 0.586. 

In Figure 4 Panel B the validate model plot is reported, and the goodness of the fit (R2 and Q2cum) is compared to one of several models based on 100 permutation validation test. This model plot shows a good predictivity of the proposed method in discriminating MuS patients from HCs. The variable important in the projection (VIP) plot, reported in Figure 4 Panel C, summarizes the most important metabolites in discriminating the two clinical groups. In the beanplots of Figure 4 Panel D the discriminant tear metabolites are shown. In particular, we found higher levels of Ser, His, Asp, C5OH/C4DC, C10:1, and C8:1 in tears from MuS patients, while lower levels of C12, C14:1, and C18:1OH were found in MuS tear samples. Quantification of Ser was also carried out on tears from 14 glaucoma patients. As shown in Supplemental Figure S1, Ser lacrimal levels are increased in MuS patients compared to both HCs and glaucoma patients. 

#### 2.3.2. Serum DIMS Analysis

**Figure 5:**
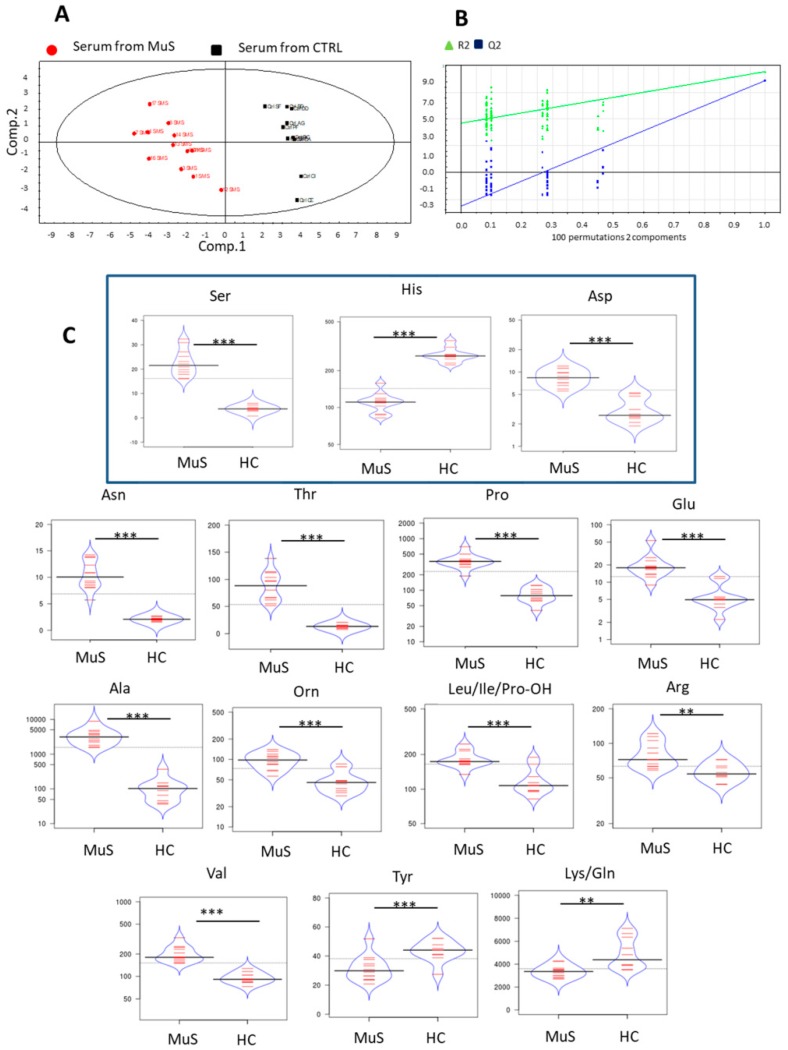
Serum metabolomics investigation by PLS-DA (**A**) Panel shows the Scores scatter plot calculated on two components. The (PLS-DA) are used to classify sera from MuS patients (symbolized as red dots) from HCs (indicated as black squares) obtained by R2Y = 0.94 and Q2(cum) = 0.861. (**B**) In Panel we report the validate model plots for serum model of PLS-DA. (**C**) The bean plots in Panel show the distribution of the serum significantly different AAs in the two analyzed groups. * means *p* < 0.05, ** means *p* < 0.01 and *** means *p* < 0.001 at the Student’s *t*-test.

The same targeted metabolomics approach, as described for tear samples, was applied for the determination of serum AAs and ACs. As for tear DIMS analysis, Table S1 also shows the mean abundance for serum AAs and ACs. In particular, we acquired sera from 12 MuS patients and 10 HCs. PLS-DA results in Figure 5 Panel A demonstrate the potential of serum metabolic profile in separating the two study groups, (R2Y = 0.94 and Q2(cum) = 0.861). Figure 5 Panel B shows the goodness of the proposed method in discriminating MuS patients from HCs, based on the metabolites reported in the bean plots (Figure 5 Panel C). Surprisingly, higher levels of Ser, Asp, Asn, Thr, Pro, Val, Ala, Orn, Leu/Ile/Pro-OH, Glu, and Arg and lower levels of His, Tyr, and Lys/Gln, were observed in MuS sera, integrating the information obtained from tears. In order to further evaluate a possible influence of sex distribution, we repeated the statistical analysis for the significantly different serum metabolites (shown in Figure 5), only considering female gender samples for both MuS patients and HCs. As reported in Table S2, our results do not show any difference, in term of significance and trend, after the exclusion of male gender in the processing. 

#### 2.3.3. Diagnostic Potential of Tear and Serum Metabolites

To verify the usefulness of tears metabolome in biomarker discovery, we compared the tear results with metabolomic data obtained from sera, a more conventional biological fluid.

**Figure 6:**
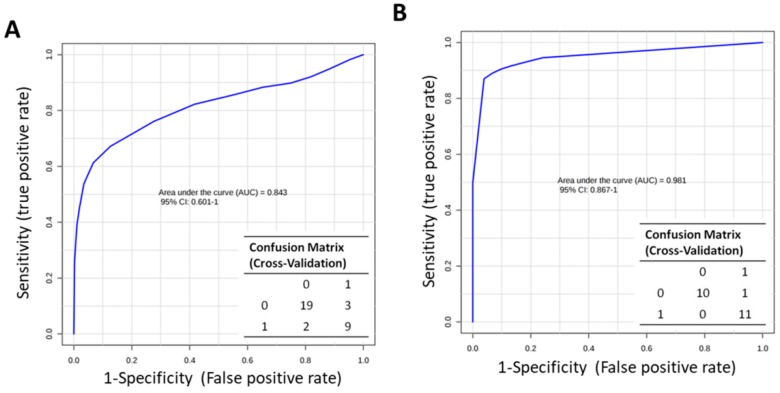
Potential Diagnostic power of tears and serum metabolites (**A**) Panel shows the Cumulative ROC curve based on tears significantly modulated ACs and Confusion Matrix obtained by 100-cross validation test using Metaboanalyst 3.0 software. Class0 = HCs and Class1 = MuS patients. (**B**) Panel shows the cumulative ROC curve and the confusion matrix based on serum levels of Ser, Thr, Asn, Glu, Orn, Tyr, and Arg. We used K-means (KM) clustering to detect the metabolites with similar behaviour to help reduce the redundancy in biomarkers analysis.

Despite the small size of samples analyzed, we tested the potential diagnostic power of tear and serum metabolites in discriminating MuS patients from HCs. The cumulative ROC curve, based on tear significantly modulated ACs, shows an AUC = 0.84. Moreover, the predicted class probability, after 100-cross validation test, highlights 82% of samples correctly classified as MuS patients (class = 1) or as HCs (class = 0) (Figure 6, Panel A). 

On the other hand, serum Ser, Thr, Asn, Glu, Orn, Tyr, and Arg show a cumulative ROC curve with an AUC of 0.98 (Figure 6 Panel B), leaving only one MuS patient not correctly classified after the 100-cross validation test. Moreover, we performed a new sample prediction analysis in the testing set of patients (18% for tear and 30% for serum sample cohort, respectively) as external validation (Figures S2 and S3). Anyway, it is important to emphasize that metabolomics data from both tear and serum analysis was obtained by a first and small clinical investigation of the integrated “omics” approach we described. Since only a small group of tear and serum samples were analyzed, it would be necessary to confirm these data in a larger and independent patient series. 

## 3. Discussion

Lipidomics and metabolomics characterization of human body fluids is becoming a useful tool for the identification of new disease biomarkers, thus improving the diagnosis and our knowledge about the molecular mechanisms involved in the disease onset [18,28]. Among the biofluids, tears potentially represent a good source of information and can be obtained through a non-invasive procedure, even if their use is still limited. However, tear potential has been exploited not only in the study of various ocular disorders, but also in the exploration of systemic disorders such as thyroid-associated orbitopathy, diabetes mellitus, and cancer [18]. Moreover, the reduced corneal sensitivity and abnormal tear function have been recently demonstrated in patients suffering from distinct neurological diseases such as Alzheimer’s disease, MuS, Parkinson’s disease, Friedreich’s ataxia, and epilepsy [29].

Therefore, following this active research field, we improved the molecular characterization of tears implementing a new experimental strategy to analyse PCs, SMs, AAs, C0 and ACs through tears on Schirmer’s strip paper. 

The use of paper strip has been already described as a collection procedure that offers an high reproducibility in terms of lipid profiles across samples [19]. Interestingly, we demonstrated that most of the analysed tear metabolites, like PC 34:1, PC 36:2 and PC 40:4, C0, C2, C3, Leu/Ile/Pro, Phe, Tyr, Glu, Met, and Lys/Gln were more retained in the first section of the imbibed paper strip. These results suggest that during the imbibition of the Schirmer’s strip, lacrimal fluid undergoes a chromatographic retention, especially for the less polar molecules, thus making the paper strip a non-homogenous sample. Therefore, it is incorrect to cut imbibed paper pieces for different extraction procedures and is preferable to use a single tear extraction of the entire Schirmer’s strip (35mm) for lipidomics and targeted metabolomics.

Once optimized, the method was applied to screen tear samples from a small set of 12 MuS patients and 21 HCs.

Lipids containing choline are recognized to play an important role in MuS [30,31], nevertheless to our knowledge no paper reports lipidomics data on MuS tears. Low levels of SMs species in MuS are the most relevant lipidomics results obtained in tears. These results are in agreement with our previous paper in which the same trend was found in MuS CSF [27]. These data are also in accordance with the literature, which describes low levels of SMs, accumulation of neurotoxic ceramides in CSF [32] and of reactive astrocytes in active lesions of MuS patients [33].

In summary, we observed 30 putative tear lipid biomarkers, five of them already found in CSF [27]. Thus, we may speculate on a possible molecular cross-talk between lacrimal fluid and CSF.

To date, only the determination of C0, C2 and C3 tear levels was reported [23,34]; in our study we were able to reveal also the medium and long-chain AC levels in such biofluid. In fact, in this pilot study, significant alterations of AC levels were observed in tears from MuS patients, suggesting a typical ACs signature. In particular, a significant increase of C5OH/C4DC, C10:1, C8:1 levels, and a significant decrease of C12, C14:1 and C18:1OH were found in tears. Surprisingly, in sera some ACs were undetectable (see supplemental Table S1), and some other results were not statistically modulated in patients. This result leads to the hypothesis that tears may represent an interesting and complementary biofluid with respect to serum, probably due to their lower molecular dynamic range of lacrimal fluid.

The AA profile in tears has been already described, either in ocular disease [35] or in the characterization of human tear metabolome [34]. Here, we revealed high tear levels of Ser, Asp and His in MuS patients. Moreover, it is intriguing to discuss the alteration observed for Ser levels in tears. Ser is involved in de novo ceramide synthesis, through a pathway catalysed by serine-palmitoyl transferase and modulated by inflammatory stimuli such as INFγ and TNFα [36], according to MuS inflammatory pathogenesis. In order to verify if the described alteration is associated to a specific modulation in MuS, Ser levels were measured also in a different pathological condition such as glaucoma. We found higher Ser levels in MuS compared to both HCs and glaucoma patients, suggesting that Ser could be associated to specific MuS events.

The quantification of serum AAs and ACs allowed the classification of MuS patients based exclusively on the levels of AAs, with a high discriminating power. In particular, Ser, His, Asp, Asn, Thr, Pro, Glu, Ala, Orn, Leu/Ile/Pro-OH, Arg, and Val, resulted higher in MuS, whereas, lower levels of Tyr and Lys/Gln were found. Interestingly, Ser levels in serum show the same trend as in tears, in accordance with its involvement in MuS inflammatory pathways. Moreover, in agreement with literature, high serum levels of Glu were found in patients, confirming its implication in neurotoxic events which occur in MuS disease [37,38,39].

## 4. Material and Methods

### 4.1. Ethics Statement

The study design was made following the guidelines for the local ethics committee that approved the study (n. 18 of 31 October 2013, protocol n. 176, Ethic committee of “G. d’Annunzio” University and ASL N.2 Lanciano-Vasto-Chieti, Italy), and conducted according to Declaration of Helsinki (World Medical Association, 1997). All patients were informed about the procedures and provided written informed consent to participate in the study. In order to protect human subject identity a number code was employed for specimen identification.

### 4.2. Patients

MuS patients, diagnosed according to the 2010 Polman’s criteria [11] were included in this study. Clinical diagnosis was confirmed by MRI studies and by the presence of OCBs in CSF. Exclusion criteria for tear collection included a history of systemic or topical therapy, ocular or systemic diseases in the previous 12 months, pregnancy, and the use of contact lens. For this pilot study 33 tear samples, from 12 MuS patients and 21 healthy subjects, and 22 serum samples (12 MuS patients and 10 HCs) were used. All patients were selected in order to obtain age, sex and ethnicity (Caucasian people) matched cohorts of subjects [4]; the clinical and demographic features of the enrolled patients are summarized in Table 2. Fourteen glaucoma patients were also enrolled for the quantification of Ser levels in tears.

### 4.3. Samples Collection

All tear samples were collected at the multiple sclerosis center of Chieti (Italy). Tear samples were collected on graduated Schirmer’s strips as previously described [16]. The Schirmer strips were purchased from EasyOpht (Busto Arsizio, Italy). Briefly, Schirmer paper strips were collected pulling the lower lid gently downward for 5 min. Then the strip was placed in a 2.0 mL Eppendorf tube and stored at −80 °C.

Sera from HCs and MuS patients were collected using Vacutainer with plain red top cup (Vacuette Tube, Greiner bio-one GmbH, Kremsmuenster, Austria for venipuncture. All samples were maintained at room temperature (23 ± 1 °C) to allow sample coagulation and centrifuged at 4 °C for 15 min at 1400× *g*. Sera has been recovered and collected in polypropylene tubes, divided into aliquots and snap frozen at −80 °C.

### 4.4. Sample Extraction

Schirmer’s strips imbibed by tears were cut into 2–3 mm paper pieces and transferred into 2.0 mL microcentrifuge tube (Eppendorf^®^, Hamburg, Germany), paying attention to wash the required equipment with MeOH before each sample preparation. Lipidomics and targeted metabolomics analysis were performed from the same Schirmer’s strip for each tear sample by the addiction of the extraction solution (PerkinElmer^®^, Turku, Finland) containing isotopically labelled internal standards (ISs) (NeoBase Non-derivatized MSMS Kit; PerkinElmer^®^, Turku, Finland). After adding 300 µL of the extraction solution containing ISs, each sample was gently mixed (50 °C, 45 min, 700 rpm) in a Thermomixer (Eppendorf^®^) and then centrifuged (13,000 rpm, 10 min). The supernatant was divided into three aliquots as follow: 100 µL for AAs and ACs determinations by DIMS analysis, 100 µL for lipidomics investigations by LC-MS/MS analysis, and the remaining volume was stored at −80 °C for further investigation.

For serum metabolite extraction, 3.2 µL of each sample were transferred into 1.5 mL tubes (Eppendorf) and then mixed with 100 µL of the same extraction solution above described, containing ISs (PerkinElmer^®^). The samples were centrifuged (15600 rpm at 4 °C for 15 min), and the supernatants were stored at −80 °C before DIMS analysis.

### 4.5. LC-MS/MS

#### 4.5.1. Choline-Containing Lipid Profiling

Lipidomics analysis was performed by using the LC-MS/MS method as already reported [40]. Briefly, the chromatographic separation of biological phospholipids was obtained by HILIC retention. Data acquisition was performed through a parent ion scan of m/z = 184 Da, which corresponds to the choline fragment, allowing the MS/MS detection of PCs, SMs and the respective Lyso-forms (LPCs and LSMs). For analytical details, see the experimental techniques section supplied in the supplementary materials. An example of the chromatogram is reported in supplemental Figure S4.

#### 4.5.2. Aminoacids and Acylcarnitines Analysis

Targeted metabolomics analysis was performed through DIMS as already reported [41,42,43]. Briefly, 30 µL of serum and tear extracted samples were injected for the determination of 20 AAs and 31 ACs. A detailed description of the analytical parameters for DIMS analysis is reported in the supplementary materials, experimental techniques section. The list of analyzed metabolites, their ionization source settings, the established functional sensitivity (µM) and their abbreviations as used in the text are available in supplementary Table S3.

### 4.6. Data Processing and Statistics

Lipidomics data were processed using MarkerLynx software (Waters, Milford, MA, USA), allowing deconvolution, alignment, and data reduction to give a table of mass and the relative retention time pairs with relative intensities for all the detected peaks. The minimum intensity considered (expressed as % BPI) was set to 10%. The data matrix obtained has been subjected to univariate statistical analysis visualized as a volcano plot using Metaboanalyst 3.0 software.

Metabolomics data were processed using NeoLynx software (Waters, UK), and levels of AAs and ACs were used to build a PLS-DA, using SIMCA-P + 11.0 software (Umetrics AB, Umea, Sweden). The D’Agostino and Pearson omnibus normality test, Student’s *t*-test, and Mann Whitney U-test were performed for comparisons between clinical groups, using GraphPad Prism (GraphPad software, Inc, La Jolla, CA 92037, USA). Bean plots were obtained by the free online BoxPlotR software http://shiny.chemgrid.org/boxplotr [44] The values of *p* < 0.05 were considered significant. The levels of significantly modulated metabolites in MuS tears were used to perform a Diagnostic power analysis by ROC curve using Metaboanalyst 3.0 software. The 95% of confidence interval was assumed for each test.

## 5. Conclusions

In conclusion, this innovative approach allows us to reveal tear PCs, SMs, C0s, ACs and AAs in a single extraction step on Shirmer’s paper, resulting in a very simple and efficient procedure for tear metabolite screening applications. In our opinion, this analytical strategy may be considered a crucial advancement in tear analysis, considering that Shirmer’s strips cutting for different extraction procedures would lead to a wrong evaluation of metabolite levels.

Furthermore, at its first application, this approach shows a great potential in characterizing even a complex pathological condition such as MuS. Despite the need of a more exhaustive study on MuS patients, our data show, for the first time, the ability of tears to deliver information that may potentially be exploited for new diagnostic perspectives of MuS. Anyway, it is worth noting that metalomics data were obtained by a first and small clinical investigation of the integrated “omics” approach, and the confirmation of these data in a larger and independent patient series would be really important.

## Figures and Tables

**Table 1:** Linear regressions performed between the levels of forty-two metabolites in thirty three Schirmer’s strip imbibed with different millimetres of tears (from 1 mm to 35 mm).

Metabolites	R Square (Linear Correlation)	Slope	Is Slope Significantly Non-Zero? *p* Value
C0	0.034	0.0001	0.298
C2	0.072	0.0001	0.129
C3	0.003	−0.0054	0.761
C4	0.093	0.0116	0.083
C5:1	0.045	0.0037	0.234
C5	0.125	0.0759	0.075
C4OH/C3DC	0.068	0.0121	0.140
C6	0.057	0.0016	0.179
C5OH/C4DC	0.090	0.0054	0.088
C5DC/C6OH	0.0001	0.0003	0.941
C8:1	0.041	0.0037	0.253
C8	0.179	0.0033	0.058
C6DC	0.005	−0.0026	0.691
C10:2	0.023	−0.0016	0.396
C10:1	0.022	0.0014	0.408
C10	0.128	0.0011	0.134
C12	0.012	−0.0010	0.531
C14:2	0.142	−0.0013	0.030
C14:1	0.190	−0.0050	0.011
C14	0.004	−0.0002	0.724
C14OH	0.136	0.0033	0.069
C18:1	0.084	0.0022	0.101
C18	0.102	0.0011	0.068
C18:1OH	0.112	−0.0035	0.056
PRO	0.056	8.3892	0.181
VAL	0.038	0.0007	0.272
LEU/ILE/PRO-OH	0.070	0.0008	0.134
ORN	0.027	4.5065	0.360
MET	0.043	0.0001	0.241
PHE	0.144	0.0005	0.072
ARG	0.297	1.0032	0.050
CIT	0.236	0.0007	0.156
TYR	0.100	0.0004	0.072
GLY	0.011	−8.1766	0.546
ALA	0.019	14.6799	0.432
SER	0.051	0.0004	0.202
THR	0.117	0.0008	0.050
ASN	0.021	0.0008	0.420
ASP	0.064	0.0003	0.155
LYS/GLN	0.023	−114.416	0.393
GLU	0.052	1.5600	0.198
HIS	0.244	2.9585	0.142

**Table 2:** Clinical and demographic features of the enrolled patients. NV = Normal Value, EDSS = Expanded Disability Status Scale, SD = Standard Deviation.

Clinical Groups	Tear Samples		Serum Samples	
	Mus	Healthy Subjects	Mus	Healthy Subjects
Number of patients	12	21	12	10
Age mean ± SD	32 ± 9	52 ± 18	41 ± 10	32.7 ± 5.5
Gender (Female%)	83%	72%	41.6%	90%
Disease phase (% of patients)	59% Stable 41% Active	--	16.6% Stable 83.3% Active	--
Link index (NV < 0.66) Range (mean ± SD)	0.48–1.6 (0.72 ± 0.3)	--	0.51–2.37 (0.88 ± 0.59)	--
CSF BOC pattern (% of patients)	83.3% Positive 16.6% not available	--	84.6% Positive 15.4% Negative	--
Barrier index (NV < 5.5) Range (mean ± SD)	2.8–9.0 (5.6 ± 2.1)	--	2.83–15.2 (8.1 ± 3.9)	--
EDSS (% of patients)	16.6% EDSS = 0 50% EDSS = 1–1.5 25% EDSS = 2–2.5 0% EDSS = 3–3.5 8.3% EDSS ≥ 4	--	25% EDSS = 1–1.5 33% EDSS = 2–2.5 25% EDSS = 3–4 8.3% EDSS ≥ 4 8.3%EDSS = n.d.	--

## Supplementary Materials

Supplementary materials can be found at https://www.mdpi.com/1422-0067/20/6/1265/s1.
