# Supplementary material for: Integrated Lipidomics and Metabolomics Analysis of Tears in Multiple Sclerosis: An Insight into Diagnostic Potential of Lacrimal Fluid
Source: Int J Mol Sci. 2019 Mar 13;20(6):1265. doi: 10.3390/ijms20061265 (PMC6471885; doi:10.3390/ijms20061265)
Supplement: Supplementary file 1 [file ijms-20-01265-s001.pdf]

Experimental Techniques section

Phospholipid profiling by LC-MS/MS analysis

The total lipid extract from tears was injected (20 μL) and separated by Alliance HT 2795 HPLC Waters Corporation). In order to obtain elution at 200 μL/min, an Atlantis HILIC silica 3μm, 150mm x 2.1mm (Waters Corporation) column was used. Separation was performed using a gradient of formic acid 0.1% (solvent A) and acetonitrile (solvent B) as follows: 92% B for 5 minutes, to 70% B in 15 minutes; isocratic 70% B for 2 minutes, to 35% B in 22 minutes; finally re-equilibration in 27 minutes. The method was developed and optimized using 8 standards of polar lipid (Avanti Polar Lipid,Inc.): DiMyristoylPhosphoCholine (DMPC), Lyso Phosphatidilcoline (14:0 LPC), Palmitoil sphingomyelin (PSM), Lyso Sphingomyelin (d18:1 LSM), DiMyristoylPhosphoEthanolamine (DMPE), Lyso Phosphoethanolamine (16:0 LPE), Distearyl-PhosphatidylSerine (DSPS), dipalmitoyl phosphatidylglycerol (DPPG) (10 μg/mL). The LC system was coupled on-line with a triple quadrupole (Quattro Ultima Platinum Micromass, Waters Corporation) through an ESI source operating in positive ion mode. A 3.5 kV tension was applied on the capillary while a 60 V tension was applied on the cone. MS/MS fragmentation functions were used to obtain the profile of biological phospholipids. Argon was used as collision gas.

Data acquisition was performed through a parent ion scan of m/z = 184 Da, which corresponds to the choline fragment, allowing the MS/MS detection of Phosphatydylcholines (PCs), Sphingomyelines (SMs) and the respective Lyso-phospholipids (LPCs and LSMs).

Amino Acids and Acylcarnitines determination by direct infusion mass spectrometry (DIMS) analysis

The DIMS analysis for the evaluation of metabolite profile in tear and serum samples was performed using a Liquid Chromatography Tandem Quadrupole Mass Spectrometry LC-MS/MS system consisting of an Alliance HT 2795 HPLC Separation Module coupled to a Quattro Ultima Pt ESI tandem quadrupole mass spectrometer (Waters Corporation). The instrument operated in positive electrospray ionization, with multiple reaction monitoring (MRM) as acquisition mode, using MassLynx V4.1 Software (Waters) with auto data processing by NeoLynx (Waters Corporation). Autosampler injections of 30 μL were made into the ion source directly by a narrow peek tube. The total run time was 1.8 minute, injection-to-injection. The mass spectrometer ionization source settings were optimized for maximum ion yields for each analyte. Capillary voltage was 3.25 kV, source temperature was 120 °C, desolvation temperature was 350 °C and the collision cell gas pressure was 3-3.5 e^{-3}mbar Argon.

Table S1. Mean abundance with the relative SD obtained from Metabolomics approach applied in tears and serum samples. n.d. = not detected.

	Tears n=33		Serum n=31	
**Metabolites**	**Mean abundance**	SD	Mean abundance	SD
**Pro**	435.7	409.8	270.3	162.3
**Ala**	202.4	130.7	3407.4	5776.3
**Arg**	53.8	34.2	73.3	23.0
**His**	245.0	193.5	161.5	79.5
**Cit**	33.5	27.7	37.9	16.7
**Gly**	1077.1	874.7	1065.0	488.4
**Leu/Ile/Pro-OH**	64.1	39.1	162.2	43.8
**Asp**	16.5	10.0	6.8	3.3
**Glu**	106.5	79.0	16.4	11.5
**Asn**	26.9	71.2	7.6	4.8
**Met**	11.7	6.7	25.3	7.2
**Orn**	212.2	141.6	82.0	39.8
**Lys/Gln**	4535.9	2839.2	3846.5	1187.7
**Phe**	33.2	18.9	43.6	9.8
**Tyr**	24.1	15.5	36.1	9.0
**Val**	24.1	17.5	167.0	62.9
**Ser**	9.8	13.5	16.7	10.2
**Thr**	6.9	10.0	59.5	37.8
**C0**	16.5	9.3	33.6	7.8
**C2**	10.6	6.7	6.5	2.0
**C3**	2.11	1.16	0.30	0.11
**C3DC/C4OH**	0.21	0.16	*n.d.*	*n.d.*
**C4**	0.66	0.44	0.17	0.07
**C5**	3.61	2.31	0.08	0.03
**C5:1**	0.26	0.15	*n.d.*	*n.d.*
**C4DC/C5OH**	0.28	0.21	*n.d.*	*n.d.*
**C6**	0.11	0.08	*n.d.*	*n.d.*
**C5DC/C6OH**	0.46	0.28	0.12	0.05
**C6DC**	0.35	0.30	0.12	0.06
**C8:1**	0.25	0.15	*n.d.*	*n.d.*
**C8**	0.21	0.12	0.13	0.08
**C10**	0.10	0.07	0.22	0.14
**C10:2**	0.20	0.12	*n.d.*	*n.d.*
**C10:1**	0.13	0.10	0.11	0.06
**C12**	0.24	0.11	0.06	0.04
**C12:1**	*n.d.*	*n.d.*	0.07	0.03
**C14**	0.12	0.05	0.03	0.01
**C14:1**	0.22	0.13	0.08	0.05
**C14:2**	0.08	0.04	*n.d.*	*n.d.*
**C14OH**	0.05	0.05	*n.d.*	*n.d.*
**C16:1**	*n.d.*	*n.d.*	*n.d.*	*n.d.*
**C16**	*n.d.*	*n.d.*	*n.d.*	*n.d.*
**C16:1-OH**	*n.d.*	*n.d.*	*n.d.*	*n.d.*
**C16OH**	*n.d.*	*n.d.*	*n.d.*	*n.d.*
**C18**	0.09	0.04	*n.d.*	*n.d.*
**C18:1**	0.08	0.04	0.11	0.04
**C18:2**	*n.d.*	*n.d.*	*n.d.*	*n.d.*
**C18:1-OH**	*n.d.*	*n.d.*	*n.d.*	*n.d.*

**C18OH**	0.17	0.12	*n.d.*	*n.d.*

Table S2. p-value of t-test and mean abundance with the relative SD obtained from serum Metabolomics approach in female people for the clinical groups considered.

Analyte	p-value	Mean Healthy Controls	Std Dev Healthy Controls	Mean MuS	Std Dev MuS
SER	8.76E-08	3.67	1.49	20.53	4.08
ASP	0.00045	3.35	1.3	6.79	1.26
HIS	5.7E-08	260.01	25.59	109.62	15.8
PRO	3.75E-06	78.86	24.91	313.48	83.94
VAL	2.16E-06	95.53	17.44	170.54	12.37
LEU/ILE/PRO-OH	0.0043	113.004	31.44	172.93	29.18
ORN	0.0013	44.99	16.79	93.04	26.8
ARG	0.0396	56.18	10.51	76.05	22.22
TYR	0.0215	43.3	7.61	32.28	7.2
ALA	0.0018	111.5	102.73	3853.26	2931.32
THR	2.39E-07	13.48	3.79	69.21	15.78
ASN	2.03E-08	2.06	0.38	9.42	1.67
LYS/GLN	0.0366	4668.5	1211.91	3413.24	772.92
GLU	0.0024	6.09	3.55	16.41	6.72

Table S3. List of 20 AAs and 31 ACs monitored, their MS ionization source settings, and their abbreviations as used in the texts.

Abbreviation IS	Full Name	Transition	Cone potential	Collision energy
Ala	Alanine	90.2>44.3	40	6
**D4Ala**		94.2>48.3		
Arg	Arginine	175.3>70.3	45	17
His	Histidine	156.2>110.2		
**D5Arg**		180.3>75.3		
Cit	Citrulline	176.2>113.2	45	13
**D2Cit**		178.2>115.2		
Gly	Glycine	76.2>30.3	40	5
**D2Gly**		78.2>32.3		
Leu/Ile/Pro-OH	Leucine/Isoleucine/Hydroxyproline	132.2>86.3	40	8
Asp	Aspartic Acid	134.2>88.2		
Glu	GlutamicAcid	148.2>84.2		
Asn	Asparagine	133.2>74.2		
**D3Leu**		135.2>89.3		
Met	Methionine	150.2>104.2	45	9
**D3Met**		153.2>107.2		
Orn	Ornithine	133.3>70.3	40	12
Lys/Gln	Lysine/Glutamine	147.2>130.2		
**D6Orn**		139.3>76.3		
Phe	Phenylalanine	166.2>120.2	45	11
**D6Phe**		172.2>126.2		
Tyr	Tyrosine	182.2>136.2	45	12
**D6Tyr**		188.2>142.2		
Val	Valine	118.2>72.3	40	8
Ser	Serine	106.1>60.3		
Thr	Threonine	120.2>74.3		
**D8Val**		126.2>80.3		
C0	Free Carnitine	162.2>103.2	60	14
**D9C0**		171.2>103.2		
C2	Acetylcarnitine	204.2>85.2	60	14

**D3C2**		207.2>85.2		
C3	Proprionylcarnitine	218.2>85.2	60	15
**D3C3**		221.2>85.2		
C4	Butyrylcarnitine	232.3>85.2		
C3DC/C4OH	Malonylcarnitine/3-Hydroxy-butyrylcarnitine	248.3>85.2		
			60	15
		235.3>85.2		
**D3C4**				
C5	Valerylcarnitine	246.2>85.2		
C5:1	Tiglylcarnitine	244.2>85.2		
C4DC/C5OH	Malonylcarnitine/3-Hydroxy-valerylcarnitine	262.2>85.2	70	16
**D9C5**		255.2>85.2		
C6	Hexanoylcarnitine	260.3>85.2	65	16
**D3C6**		263.3>85.2		
C5DC/C6OH	Glutarylcernitine/3-Hydroxy-	276.3>85.2		
	hexanoylcarnitine	290.3>85.2	70	20
C6DC	Adiylcarnitine			
**D6C5DC**		282.3>85.2		
C8:1	Octenoylcarnitine	286.3>85.2		
C8	Octanoylcarnitine	288.3>85.2	75	18
**D3C8**		291.3>85.2		
C10	Decanoylcarnitine	316.3>85.2		
C10:2	Decadienoylcarmitine	313.3>85.2	75	19
C10:1	Decenoylcarnitine	314.3>85.2		
**D3C10**		319.3>85.2		
C12	Dodecenoylcarnitine	344.4>85.2		
C12:1	Dodecanoylcarnitine	342.4>85.2	75	22
**D3C12**		347.4>85.2		
C14	Tetradecanoylcarnitine	372.4>85.2		
C14:1	Tetradecenoylcarnitine	370.4>85.2		
C14:2	Tetradecadienoylcarnitine	368.4>85.2	75	23
C14OH	3-Hydroxy-tetradecanoylcarnitine	388.4>85.2		
**D3C14**		375.4>85.2		
C16:1	Hexadecenoylcarnitine	398.4>85.2		
C16	Hexadecanoylcarnitine	400.4>85.2		
C16:1-OH	3-Hydroxy-hexadecenoylcarnitine	414.4>85.2	75	25
C16OH	3-Hydroxy-hexadecanoylcarnitine	416.4>85.2		
**D3C16**		403.4>85.2		
C18	Octadecanoylcarnitine	428.4>85.2		
C18:1	Octadecenoylcarnitine	426.4>85.2		
C18:2	Octadecadienoylcarnitine	424.4>85.2	80	25
C18:1-OH	3-Hydroxy-octadecenoylcarnitine	442.4>85.2		
C18OH	3-Hydroxy-octadecanoylcarnitine	444.4>85.2		
**D3C18**		431.4>85.2		

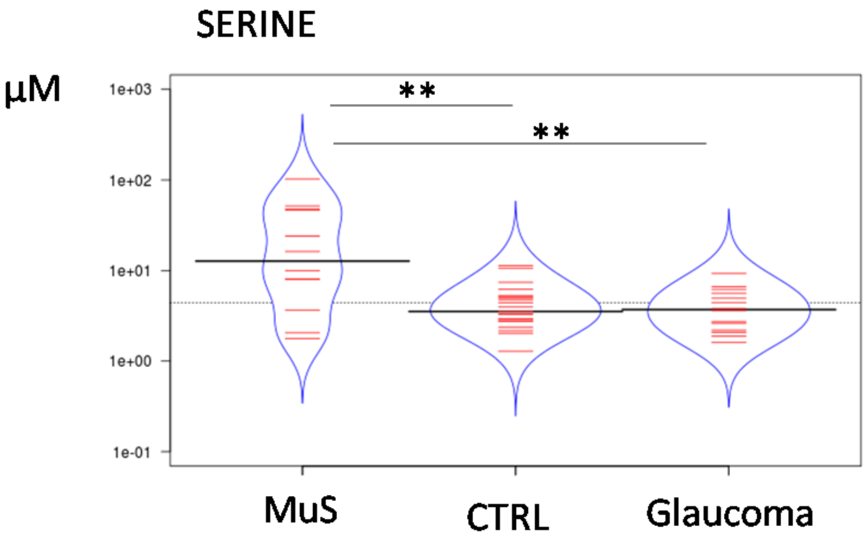

Figure S1. Figure shows the bean plots of Serine concentration in tears from MuS patients, healthy people (CTRL) and patients with Glaucoma. ** means $p<0.01$ at the Student's t-test or Mann Whitney U-test.

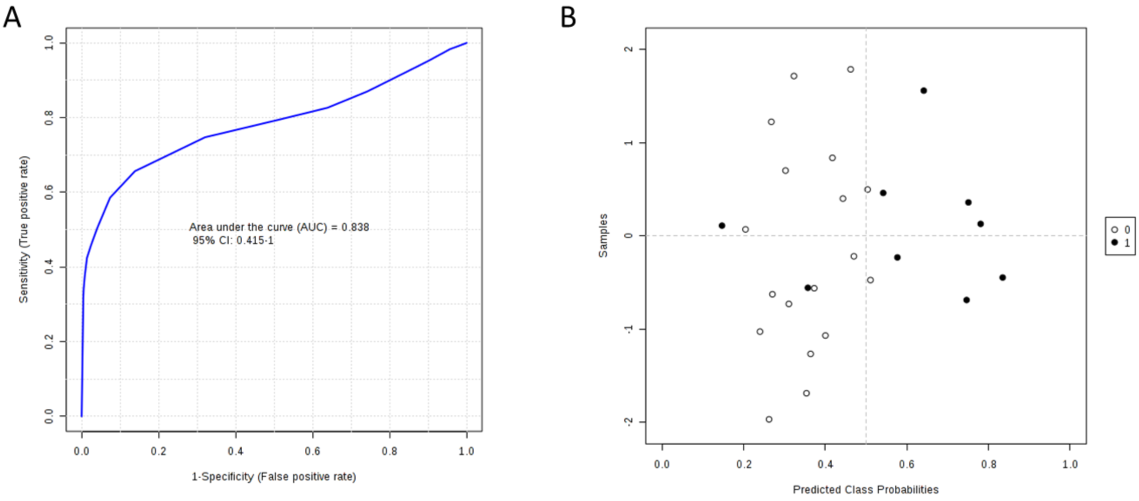

C

Confusion Matrix (Cross-Validation)		
	0	1
0	16	2
1	2	7

D

Name	Probability	Class
04_MuS	0.83154	1
05_MuS	0.56091	1
12_MuS	0.69296	0
04_HC	0.60005	0
08_HC	0.71379	0
11_HC	0.51492	1

Figure S2. In Figure we report a combine ROC curve based on significantly different tear ACs, in a training set of patients (82%), as well as for external validation purpose, we report the predicted diagnosis for new samples (testing set, 18%). In Panel A, combined ROC curve with an AUC of 0.838 is reported. Panel B shows the predicted class probabilities of each sample through the 100-cross validation, underlying good predictivity of the proposed model in discriminating MuS patients from

Healthy controls, as reported in the confusion matrix in Panel C. As model external validation, we performed a new sample prediction analysis in the testing set of patients, showing that two patients out of three are correctly classified (Panel D). Class 0= Healthy Controls. Class 1= Multiple Sclerosis patients.

A B

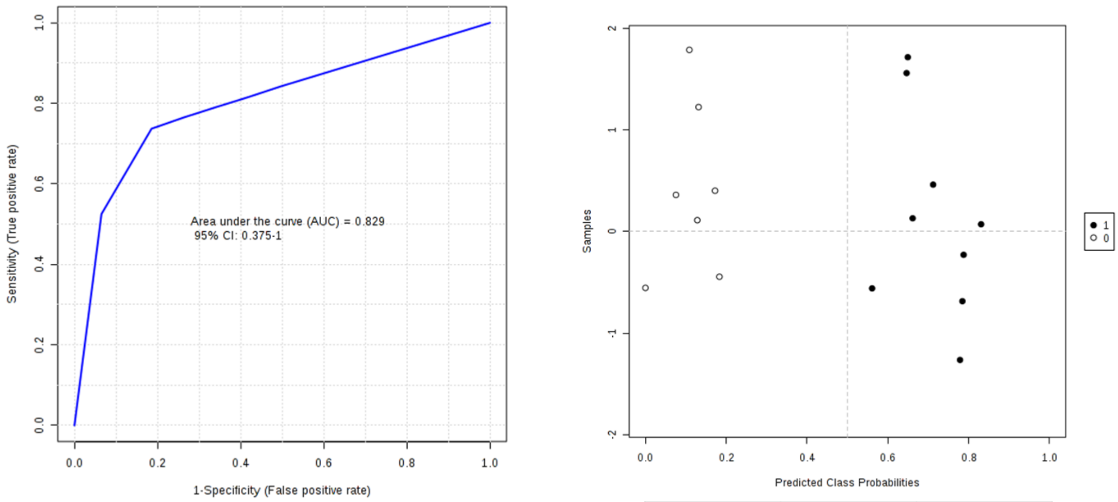

C

Confusion Matrix (Cross-Validation)		
	0	1
0	7	0
1	0	9

D

Name	Probability	Class
01_HC	1.0	0
02_HC	1.0	0
03_HC	1.0	0
08_MuS	0.99999	1
12_MuS	1.0	1
01_MuS	1.0	1

Figure S3. In Figure we report a combine ROC curve, calculated on the modulated AAs in serum from HC and MuS, in a training set of patients (70%), as well as for external validation purpose, we report the predict diagnosis for new samples (testing set, 30%). In Panel A Combined ROC curve with an AUC of 0.829 is reported. Panel B shows the predicted class probabilities of each sample through the 100-cross validation, underlying good predictivity of the proposed model in discriminating MuS patients from healthy controls, as reported in the confusion matrix in Panel C. As model external validation, we performed a new sample prediction analysis in the testing set of patients, showing that all patients are correctly classified(Panel D). Class 0= Healthy Controls. Class 1= Multiple Sclerosis patients.

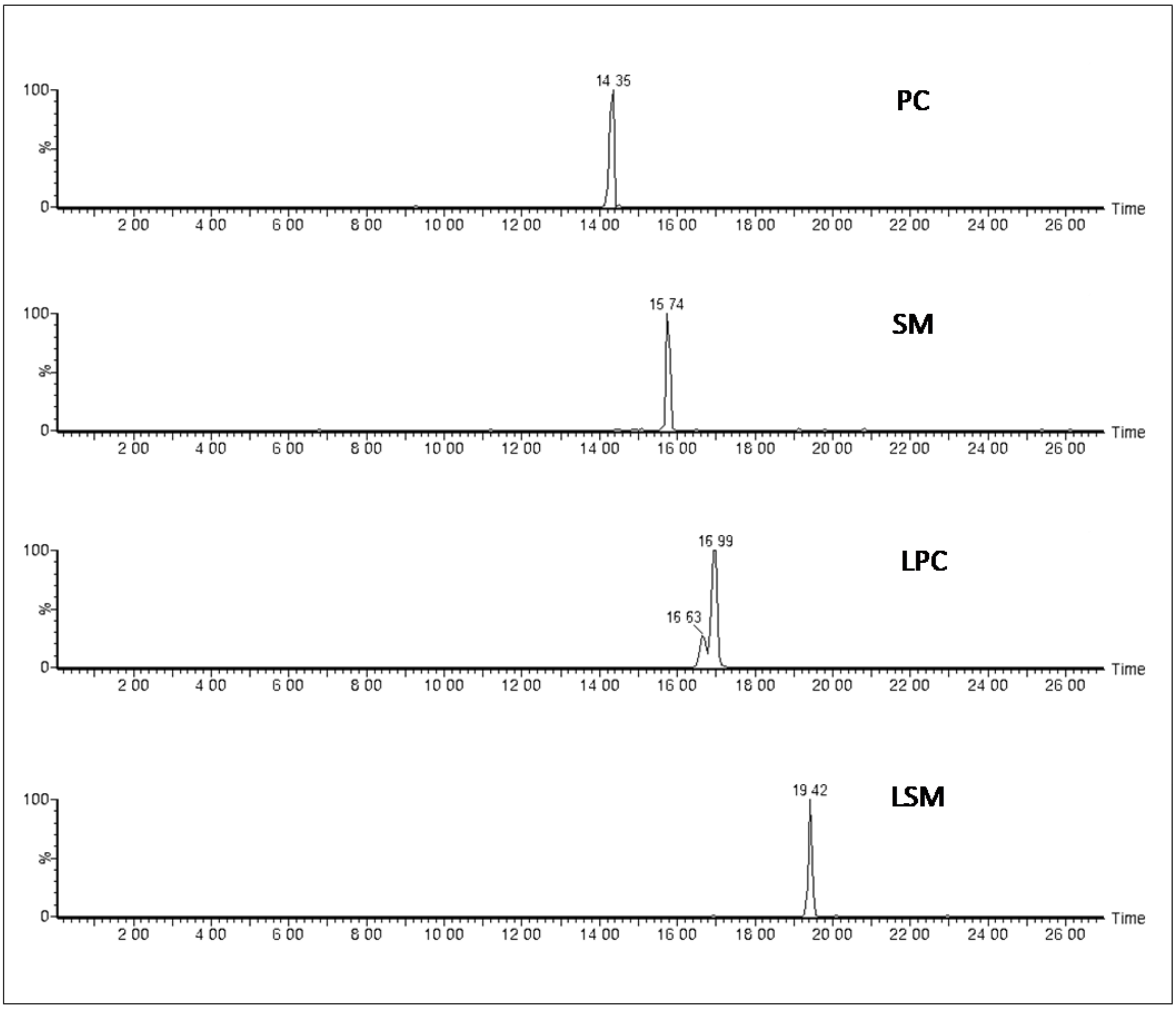

Figure S4. Example of Chromatogram for PC, LPC and SM LC-MS/MS determination.
